# Effects of Motivational Interview-Based Cognitive Therapy in Patients With Schizophrenia: A Multicenter Randomized Controlled Trial

**DOI:** 10.1097/jnr.0000000000000761

**Published:** 2026-08-13

**Authors:** Yan-Liang CHEN, Chieh-Yu LIU, Chiu-Yueh YANG

**Affiliations:** 1 College of Nursing, National Yang Ming Chiao Tung University, Taipei, Taiwan; 2 School of Nursing, Yuan Ze University, Taoyuan, Taiwan; 3 Institute of Community Health Care, College of Nursing, National Yang Ming Chiao Tung University, Taipei, Taiwan; 4 Institute of Clinical Nursing, College of Nursing, National Yang Ming Chiao Tung University, Taipei, Taiwan

**Keywords:** cognitive therapy, insight, medication adherence, motivational interviewing, schizophrenia

## Abstract

**Background:**

Nonadherence to treatment increases relapse and rehospitalization risks and diminishes quality of life in patients with schizophrenia spectrum disorder.

**Purpose:**

This study was developed to explore the effect of motivational interview-based cognitive therapy (MIBCT) on medication adherence, insight into illness, and psychotic symptom severity in patients with schizophrenia spectrum disorder.

**Methods:**

A single-blind, multicenter randomized controlled trial was conducted on 120 participants recruited from outpatient departments and acute psychiatric wards in northern Taiwan. The participants were randomly assigned to either the experimental group (*n*=61), which received MIBCT consisting of eight sessions every 3–4 weeks over a period of 24–32 weeks, or the control group (*n*=59), which received routine care and psychoeducation. The outcomes were measured using the Medication Adherence Rating Scale (MARS), the Schedule Assessment of Insight (SAI), and the Positive and Negative Syndrome Scale (PANSS) at three time points: baseline, the fourth session of interim analysis, and postintervention. Differences in between-group outcomes over time after adjusting for 13 covariates were analyzed using generalized estimating equations.

**Results:**

The mean MARS score in the experimental group improved significantly by 2.56 points at postintervention (*p*<.001), with significantly lower gains reported for the control group. In addition, the SAI outcome measures for the experimental group improved more than for the control group. This was evident both during the interim analysis and postintervention, with results of *B*=1.85 (*p*=.003) and *B*=2.23 (*p*=.001), respectively. Furthermore, the mean improvements in positive symptoms were significantly greater in the intervention group than the control group at both the interim analysis (*B*=−2.52, *p*=.002) and postintervention (*B*=−1.85, *p*=.029).

**Conclusions/Implications for Practice:**

The results of this study indicate the application of MIBCT significantly improves medication adherence, insight into illness, and positive symptoms in patients with schizophrenia spectrum disorders by increasing intrinsic motivation and addressing treatment barriers. The implementation of this integrated therapeutic approach by psychiatric nurses may contribute to improved patient outcomes.

## Introduction

Schizophrenia, a chronic psychotic disorder that affects ~1% of the global population, is characterized by symptoms such as delusion, disorganized speech, hallucination, and impaired executive functioning. Without effective treatment, schizophrenia may lead to substantial disabilities in social, occupational, and daily functioning, resulting in reduced quality of life (Jauhar et al., 2022).

Medication adherence presents a significant challenge in the treatment of schizophrenia spectrum disorders. Antipsychotic medications are the primary means of preventing relapse (Schneider-Thoma et al., 2022). However, many patients lack insight into their illness, which leads to poor medication adherence (Nyanyiwa et al., 2022), associated with worsening symptoms, increased risks of relapse and rehospitalization (Maramis et al., 2021), and even suicide (Warriach et al., 2021).

Approximately 58.2% of patients stop taking their antipsychotic medication within 6 months of hospital discharge (Vega et al., 2021). This high rate of nonadherence is a persistent and significant issue in psychiatric care. As consistent and continuous medication is essential for maintaining clinical stability, the complex nature of adherence behavior poses ongoing challenges for both patients and health care providers (Araya et al., 2020; Deng et al., 2022).

Nonadherence in patients with schizophrenia involves multifactorial causes across the following three dimensions: personal-physiological, which includes patients’ experiences of medication side effects (Hsieh et al., 2019; Semahegn et al., 2020); psychological, which encompasses attitudes toward illness, self-efficacy, stigma, and clinical insight (Deng et al., 2022; Semahegn et al., 2020); and social-environmental, which involves therapeutic alliances, support systems, and societal stigma that influence adherence behaviors (Hsieh et al., 2019; Semahegn et al., 2020). Better understanding these dimensions may be expected to assist researchers and health care providers to more effectively address the factors contributing to nonadherence in patients with schizophrenia.

Although several psychosocial interventions have been developed to improve adherence (Cahaya et al., 2022), many overlook the complex interplay among the factors involved. For example, the family psychoeducation intervention including pharmacological and cognitive components implemented by Tessier et al. (2023) in France not address internal processes such as ambivalence and insight. Similarly, Turkish scholars Kızılırmak Tatu and Demir (2021) focused on awareness and attitudes but neglected important relational and cognitive mechanisms such as therapeutic alliances and stigma.

Motivational interview-based cognitive therapy (MIBCT) is a structured psychological intervention designed to improve medication adherence in patients with schizophrenia. By combining motivational interviewing (MI) techniques, which increase intrinsic motivation and reduce ambivalence (Miller & Rollnick, 2023), with cognitive strategies such as Beck’s cognitive therapy (CT), which focuses on changing maladaptive beliefs and boosting self-efficacy (Iarussi, 2020), MIBCT comprehensively addresses the primary barriers to adherence by encouraging patients to evaluate their beliefs, recognize the benefits of medication, and confront their internalized stigma. Moreover, MIBCT facilitates the creation of a robust therapeutic alliance and facilitates discussions on illness management to increase social support and reduce stigma (Chien et al., 2016; Gray et al., 2010).

The complexity of nonadherence in schizophrenia spectrum disorders requires a comprehensive approach, as no single intervention has consistently achieved strong outcomes. In previous research, an MIBCT, aka compliance/adherence therapy (CAT), intervention was implemented in Thailand that combined motivational interviewing with cognitive behavioral therapy and psychoeducation using a collaborative, client-centered approach to strengthen commitment to treatment (Inwanna et al., 2022; Wong-Anuchit et al., 2019).

Despite the advances made, prior research on this subject has differed significantly in terms of total session number (5–8), session duration (60–90 min), and treatment intensity. In addition, many of these studies do not report direct outcomes related to medication adherence or insight into illness (Inwanna et al., 2022; Wong-Anuchit et al., 2019). Furthermore, the results of a recent systematic review highlighted considerable heterogeneity in motivational interviewing studies, with session durations ranging from six sessions over periods of 6–26 weeks and follow-up periods ranging from 2 weeks to 18 months (Cahaya et al., 2022).

Chien et al. (2016) originally developed MIBCT to improve medication adherence in individuals with schizophrenia spectrum disorders. This structured intervention combines MI techniques with cognitive strategies to address maladaptive beliefs. Although MIBCT aims to increase medication knowledge and overcome adherence barriers, limited focus is given to insight into the illness and inpatients and patients on long-acting injectable medications are excluded. Moreover, MIBCT fails to fully consider crucial factors such as antipsychotic dosage and internalized stigma. Therefore, the aim of this study was to examine how MIBCT can improve medication adherence during the important period of time when patients shift from hospital to community care. Therapy measures start to be administered during the later days of hospitalization and continue after discharge. This approach helps maintain treatment and encourage adherence. In this study, important gaps in the current literature are addressed by focusing on the psychological and cognitive aspects of medication adherence using an eight-session MIBCT program designed to address intrinsic motivation, insight, cognitive distortions, stigma, and social support.

Because MIBCT is a theory-based, patient-centered approach that meets the real-world needs of patients with schizophrenia spectrum disorders, especially during transitions in care, using it to improve medication adherence may be expected to help prevent relapses, support ongoing recovery, and reduce rehospitalization rates (Iarussi, 2020).

### Purpose and Hypotheses

This study was designed to examine the effects of MIBCT on medication adherence, insight into illness, and psychotic symptom severity in patients with schizophrenia spectrum disorder. The two hypotheses of this study were that the experimental group (EG) will show significantly greater improvements than the control group (CG) in terms of:

1. Participants receiving MIBCT will demonstrate significantly improved medication adherence at both the interim assessment (fourth intervention session) and postintervention compared with baseline.
2. Insight into illness and reduced psychotic symptom severity compared with baseline both at the fourth session after the interim analysis and at postintervention.

## Methods

### Research Design

This was implemented as a multicenter randomized controlled trial with a single-blind study design. Convenience sampling and blocked randomization with a block size of 4 following a 1:1 allocation ratio were used. A list of random numbers and corresponding group assignment codes was generated using random allocation software and sealed in opaque envelopes beforehand by the third author, who was not involved in participant recruitment or intervention implementation. The three data collectors, who also served as outcome assessors, were unaware of the group assignments. After the baseline data were collected, the first author implemented the intervention. The primary outcome was measured using the MARS. The secondary outcomes were SAI score and PANSS score. The EG participated in eight 50–60-min MIBCT sessions, whereas the CG received routine care only in eight sessions of 50–60 min each. The participants were assessed at baseline (T_0_), at the fourth session after the intervention interim analysis (T_1_), and at postintervention (T_2_). All clinical assessments of the participants were performed by blinded research assistants.

### Participants, Setting, and Recruitment

In Taiwan, patients admitted to acute psychiatric wards are typically informed of their discharge date 1–2 weeks in advance. During their time in the hospital, nurses begin preparing their post-discharge care plans. The researchers were involved in selecting potential participants from among those qualified patients approaching the end of their acute care.

In alignment with discharge preparations, recruitment focused on those patients scheduled for discharge within 1–2 weeks. Initially, the ward nurse identified suitable candidates and evaluated their willingness and eligibility to participate. After the completion of pretesting, the intervention was initiated ~1 week before discharge, that is, the first MIBCT session was conducted while participants were inpatients.

Follow-up care was coordinated with the participants’ regular outpatient appointments, which, in Taiwan, are usually scheduled at 3–4-week intervals, giving this study a follow-up period of between 24 and 32 weeks.

One hundred and twenty patients diagnosed with DSM-5 criteria-based schizophrenia spectrum disorder were recruited and enrolled as participants. The inclusion criteria were: (1) 20–64 years old; (2) clinical diagnosis of schizophrenia and schizoaffective disorder according to the Diagnostic and Statistical Manual of Mental Disorders (5th edition) made by an independent psychiatrist; (3) Chinese reading and writing skills; (4) family member or health care staff reports indicating the patient does not take medicine regularly or otherwise reduces the dosage or adjusts the frequency of their antipsychotic drug use; and (5) willingness to sign the consent form. The exclusion criteria were: (1) comorbidities including intellectual disturbance, organic brain syndrome, cognitive impairment, or substance abuse; (2) psychotic symptom disturbances that make the patient effectively unable to communicate with others; and (3) participation in a medication treatment management program during the current hospitalization or within the previous month. This study was conducted between October 2018 and September 2019. The participants were recruited from two psychiatric outpatient departments and six acute wards at two psychiatric teaching hospitals in northern Taiwan.

### Sample Size

The minimum sample size of this study was calculated as 86 (with the assumption of a 30% attrition rate) with G-Power 3.1.9.2 using a repeated-measures analysis of variance. The effect size was *f*=0.25, as suggested by Cohen, with a power of 0.8, an α of .05, a number measurement of 3, and a correlation coefficient of .50. One hundred twenty participants completed valid questionnaires at all three time points (T_0_–T_2_). Cohen d, a measure of effect size, was calculated for the interaction between group and time by comparing change in scores using a *t* test. The *t* statistic was then converted to Cohen d by using the formula *d* = 2(*t*)/sqrt(*df*), with *df* denoting degrees of freedom.

### Interventions

Those participants in the CG received medication, psychoeducation, and eight regular interview sessions. Each interview session lasted ~30–50 min and involved discussions of methods of disease coping and the importance of taking medications regularly and as directed.

Those participants in the EG received eight sessions of MIBCT. The intervention was based on Gray et al. (2010) and Chien et al. (2016) and is a form of psychoeducation based on motivational interviewing and cognitive behavioral therapy. The individual face-to-face interventions were administered in eight sessions over a 24–32-week period. Intervention duration varied based on whether the individual participant had their regular outpatient clinic follow-up scheduled at 3 or 4-week intervals. Each intervention lasted 30–50 min on average. The first session was held in either an outpatient clinic or acute care ward, with the remainder held in outpatient clinics.

After consent was obtained from both WT Chien and R. Gary, the content of the MIBCT intervention was adapted to better align with Taiwan’s cultural context. The cultural adaption of the content was evaluated by a panel of five experts, which included a psychiatrist, a nurse educator, and three senior psychiatric mental health nurses, who discussed the relevance and feasibility of the content until all experts reached strong consensus on appropriateness.

The MIBCT sessions were conducted in designated consultation or treatment rooms within either an outpatient department or inpatient ward to ensure participant privacy and minimize disruptions. Although in the acute ward, the participants received the intervention during nonroutine treatment times. As outpatients, the participants received the intervention during their waiting periods. After each intervention session, the next appointment time was agreed upon by both the patient and his or her family on the basis of the appointment schedule.

The psychiatric nurse (YLC) delivered the MIBCT program using face-to-face individual interviews that included three components: (1) providing comprehensive insights into the illness of patients with schizophrenia and corresponding pharmacological interventions to facilitate instruction on self-management skills; (2) clarifying patient goals for change and assisting in formulating a plan for taking antipsychotics; and (3) evaluating the patient’s medication adherence and insights into their illness. A summary of the eight MIBCT sessions is provided in the Appendix, Supplemental Digital Content 1, https://links.lww.com/JNR/A17.

The MIBCT intervention was conducted by a master’s student in nursing with 3.5 years of psychiatric nursing experience and certification in motivational interviewing (MI). The intervener had completed a 24-hr MI training program with supervision from a certified specialist and had 288 hr of advanced community nursing practice and 144 hr of advanced psychiatric practice. As part of preparation for this study, the intervener and academic supervisor conducted a case study on a patient with schizophrenia and applied MI techniques to improve skills and clinical relevance.

In the first phase of this study, both CG and EG participants completed a baseline assessment while in outpatient clinics or acute psychiatric wards. Throughout the study, all of the participants continued to receive standard care from their psychiatrists.

### Instrument

Sociodemographic data and Internalized Stigma of Mental Illness Scale (ISMIS) score were used in this study as the control variables. The primary outcome indicator was MARS score, whereas the secondary outcomes were SAI and PANSS scores.

#### Sociodemographic Data

The baseline assessment of sociodemographic data included sex, age, years of education, marital status, living conditions, disease diagnosis, work status, disease duration, medication type, and chlorpromazine-equivalent dose (Lin et al., 2018).

#### Internalized Stigma of Mental Illness Scale (ISMIS)

The ISMIS, developed to assess the degree of patient self-stigmatization regarding their mental illness, includes 29 questions scored on a four-point Likert scale, with higher scores associated with higher degrees of self-stigmatization. Chang et al. (2014) translated and verified the traditional Chinese version used in this study, with a Cronbach α of .94 and intraclass correlation coefficient (ICC) of test-retest reliability of .78. Concurrent validity testing revealed the physical symptoms of the Depression and Somatic Symptoms Scale to correlate positively with each subscale of the ISMIS. In this study, the Cronbach alpha was .928, indicating high internal consistency.

#### Primary Outcome: Medication Adherence Rating Scale (MARS) Score

The MARS was developed by Thompson et al. (2000) as a self-report scale used to evaluate patient attitudes toward medication taking and adherence behavior within the past week. The MARS includes ten items with yes/no responses, with 0 indicating poor medication adherence 1 indicating good medication adherence. The total scale score ranges from 0 to 10. The Chinese version of the MARS for Taiwan has an internal consistency of 0.72 and a retest reliability of .8 (Kao & Liu, 2010).

#### Secondary Outcome: Schedule for the Assessment of Insight (SAI)

The SAI was developed by David (1990) to measure patient insight and need for treatment and to help interpret symptoms. The SAI includes seven items scored between 0 and 2, with higher scores indicating better patient insight into their illness. The SAI is interviewer-scored based on semi-structured interviews. The total scale score range from 0 to 14 points. The traditional Chinese version used in Taiwan has a Cronbach α of .72, a correlation coefficient of .92, and good construct validity and concurrent validity (Yen et al., 2008). Inter-rater reliability was assessed for this study, with the ICC of .92 indicating good consistency and agreement. The Cronbach alpha was .870 in this study.

#### Secondary Outcome: Positive and Negative Syndrome Scale (PANSS) Score

The 30-item PANSS developed by Kay et al. (1987) assesses the severity of the psychotic symptoms of mental illness. It is based on semi-structured interviews and is scored on a seven-point Likert scale based on symptom severity, with higher scores reflecting greater symptom severity. The total possible scale score ranges from 0 to 210. The Chinese version was verified by five Taiwanese specialists on 30 patients with acute and chronic schizophrenia, with an ICC of 66.7% (Cheng et al., 1996). In this study, the Cronbach alpha was .903 and the measured inter-rater reliability was .92.

### Data Collection

Data for this study were collected from October 2018 to September 2019. The psychiatric professionals responsible for the participants’ community care were all informed regarding the study objectives and content. After the participants signed the consent form, data collection for the SAI and the PANSS was conducted by three research assistants, each of whom had more than 5 years of experience in psychiatric nursing. During the individual face-to-face interviews, if the participant’s symptoms impacted the interview process, the interview was terminated with the participant’s consent. Eligible participants were identified either by psychiatric nurses or through a review of medical records. The sociodemographic and measured data collected were used solely for this study. The data were collected at three time points: baseline (T_0_), at the fourth session after the intervention interim analysis (T_1_), and at postintervention (T_2_). A structured questionnaire was used to collect data for the MARS, SAI, and PANSS.

During data collection at the outpatient clinic, patients arrived individually for their scheduled medical appointments and waited quietly outside the physician’s office because the facility did not have a designated waiting room. The lack of opportunity in this setting for patients to interact with one another minimized the risk of between-group cross-contamination.

### Statistical Analysis

Data analysis was performed using IBM SPSS Statistics 24.0 for Windows (IBM Corp., Armonk, NY, USA). Significance level and frequency, percentage, mean, *SD*, and other methods were used to describe participant demographic and clinical characteristics. The χ^2^ test was used to detect the homogeneity of the categorical data for the demographic and clinical characteristics of the two groups. Independent *t* tests were used to detect differences between continuous variables for demographic and clinical characteristics and outcome indicators between the EG and CG. The generalized estimating equation (GEE) of Liang and Zeger (1986) was used to analyze between-group and within-group changes in outcome indicators in the EG. GEE was used to control the impact of demographic and clinical characteristics on the outcome variables and to test changes in the two groups of participants over time and the degree of improvement in outcome indicators.

### Research Ethics

Participant recruitment commenced after approval from the institutional review boards of Bali Psychiatric Teaching Hospital (No IRB 1070910-3) on October 1, 2018; Tri-Service General Hospital (No IRB 2-107-05-116) on August 29, 2018; and National Yang-Ming University (No IRB YM108024E) on February 27, 2019. Before enrollment, all potential participants were fully informed of the study’s purpose, procedures, and potential risks, and written informed consent was obtained from each. Throughout the study period, the participants retained the right to withdraw at any time without penalty. All of the participants were clinically stable, cognitively intact, and fully alert during the informed consent process and already scheduled for hospital discharge. If any symptoms or discomfort arose during the study, psychiatric professionals were available to provide timely and appropriate care.

When participants were accompanied by their primary caregiver during outpatient follow-up visits, these caregivers were informed of the study and made aware of their family member’s participation. In cases where caregivers raised concerns or objected to the participant’s involvement, they were encouraged to discuss the matter directly with the participant. If necessary, caregivers could request the participant’s voluntary withdrawal from the study in line with the ethical principles of autonomy, respect, and shared decision-making.

## Results

### Participant Sociodemographic and Clinical Characteristics

**Figure 1:**
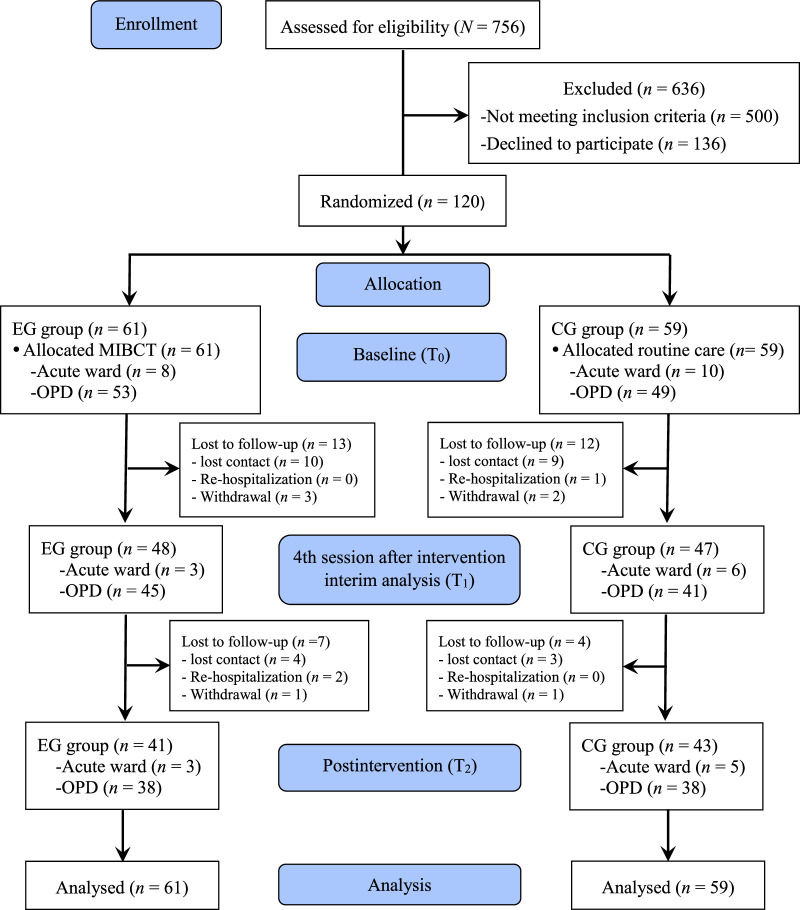
Consolidated standards of reporting trials flow diagram *Note.* CG = control group; EG = experimental group; MIBCT = motivational interviewing-based cognitive therapy; OPD = outpatient department.

A total of 756 participants were screened based on the enrollment criteria, with 120 meeting these criteria. The EG included 61 participants and the CG included 59 participants. The demographic information and outcome variables for the two groups are shown in Table 2. In terms of the sample, the average age was 45.67 (*SD*=10.46) years, average number of years of education was 11.55 (*SD*=2.54) years, and average disease duration was 19.69 (*SD*=12.04) years. The attrition rate was 21% (*n*=25) in the fourth session after the intervention interim analysis (T_1_) and 9% (*n*=11) at postintervention (T_2_). The study data were analyzed according to the intention-to-treat (ITT) principle, with the procedure performed according to the Consolidated Standards of Reporting Trials (CONSORT) checklist standard, the flow diagram for which is given in Figure 1. No significant differences in sociodemographic data, clinical variables, or outcome indicators between the two groups were identified at baseline (Table 1).

**Table 1:** Demographic Data of the Experimental and Control Groups at Baseline (*N*=120)

Variable	CG (*n*=59)		EG (*n*=61)		χ^2^	*p*
	*n*	*%*	*n*	*%*		
Sex					0.800	.371
Female	26	44.1	22	36.1		
Male	33	55.9	39	63.9		
Diagnosis						1.313	.252
Schizophrenia	49	83.1	55	90.2		
Schizoaffective disorder	10	16.9	6	9.8		
Marital status							1.450	.266
Single	50	84.7	56	91.8		
Married	9	15.3	5	8.2		
Working status						3.082	.079
Employed	17	28.8	27	44.3		
Unemployed	42	71.2	34	55.7		
Treatment setting						0.346	.615
Acute ward	10	16.9	8	13.1		
Outpatient department	49	83.1	53	86.9		
Living alone						0.688	.406
Yes	11	18.6	8	13.1		
No	48	81.4	53	86.9		
Type of antipsychotics						0.965	.326
Oral	44	74.6	50	82		
Oral and depot	15	25.4	11	18		
Antipsychotics						1.256	.534
Typical	11	18.6	7	11.5		
Atypical	42	71.2	48	78.7		
Combine	6	10.2	6	9.8		

Variable	Mean	*SD*	Mean	*SD*	*t*	*p*
Age	45.05	11.15	46.26	9.81	0.148	.528
Years of formal education	11.47	2.25	11.62	2.81	−0.319	.751
Duration of disease	19.51	14.07	19.87	9.81	−0.163	.871
ISMIS	2.60	0.45	2.48	0.46	1.484	.141
CPZ _(100)_ equivalent dose	412.19	282.83	431.27	241.21	−0.398	.961
MARS	5.31	2.91	4.52	2.69	1.526	.130
SAI	6.51	2.97	6.26	2.85	0.463	.644
PANSS	84.54	16.30	82.95	17.32	0.518	.605

*Note.* CG = control group; CPZ = chlorpromazine; EG = experimental group; ISMIS = Internalized Stigma of Mental Illness Scale; MARS = Medication Adherence Rating Scale; PANSS = Positive and Negative Syndrome Scale; SAI = Schedule for the Assessment of Insight.

### Effects of the MIBCT Intervention

**Figure 2:**
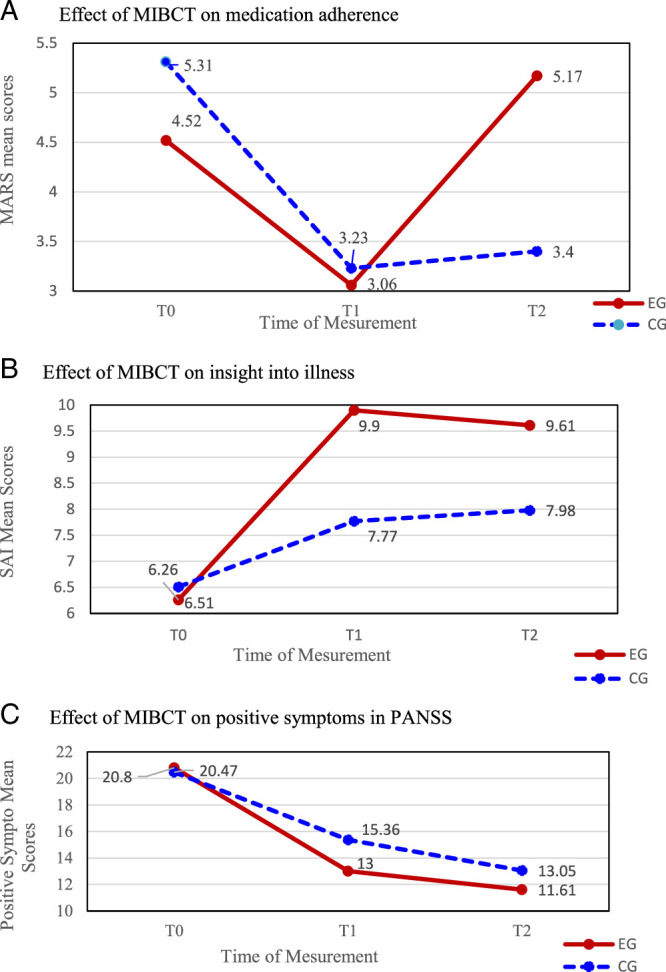
Between-group comparison of effects of motivational interviewing-based cognitive therapy (MIBCT) on outcome changes (*N*=120) *Note.* Mean scores of outcome indicators for the experimental group and control group at baseline (T0), interim assessment (T1; fourth intervention session), and postintervention (T2). MARS = Medication Adherence Rating Scale; PANSS = Positive and Negative Syndrome Scale; SAI = Schedule for the Assessment of Insight.

Three outcome indicators were used in this study, with the results for each outcome shown in Figure 2 and within and between-group differences in change over time shown in Table 2. The results of the GEE controlling for the effects of 13 control variables are also given in Table 2.

**Table 2:** Effects of Motivational Interviews-Based Cognitive Therapy (MIBCT) on Medication Adherence, Insight into Illness and Psychotic Symptoms at the Three Time Points (T_0_–T_2_; *N*=120)

Primary Outcome: Medication Adherence (MARS)						
Setting/Variable	EG (*n*=61)			CG (*n*=59)		
	T_0_	T_1_	T_2_	T_0_	T_1_	T_2_
	*M* (*SD*)	*M* (*SD*)	*M* (*SD*)	*M* (*SD*)	*M* (*SD*)	*M* (*SD*)
All settings	4.52 (2.69)	3.06 (2.53)	5.17 (2.32)	5.31 (2.91)	3.23 (2.00)	3.40 (1.81)
Acute ward	5.38 (3.34)	7.00 (2.65)	7.00 (1.00)	5.40 (1.90)	3.33 (2.50)	2.80 (0.84)
OPD	4.40 (2.59)	2.80 (2.32)	5.03 (2.34)	5.29 (3.10)	3.22 (1.96)	3.47 (1.90)

Setting/Effect	*B*	*SE*	95% CI		*p*	*ES*
			Lower	Upper		
All settings						
Group	−0.54	0.48	−1.48	0.40	.259	
T_1_	−2.01	0.42	−2.83	−1.19	<.001	
T_2_	−1.86	0.43	−2.70	−1.01	<.001	
Group×T_1_	0.65	0.60	−0.53	1.83	.281	0.221
Group×T_2_	2.56	0.61	1.36	3.76	<.001	0.912
Acute ward						
Group	−0.03	1.24	−2.46	2.41	.984	
T_1_	−2.07	1.10	−4.22	0.09	.060	
T_2_	−2.60	0.66	−3.89	−1.31	<.001	
Group×T_1_	3.69	2.04	−0.30	7.68	.070	0.739
Group×T_2_	4.23	1.54	1.20	7.25	.006	1.179
OPD						
Group	−0.89	0.56	−1.99	0.21	.113	
T_1_	−2.07	0.46	−2.96	−1.17	<.001	
T_2_	−1.81	0.48	−2.74	−0.88	<.001	
Group×T_1_	0.47	0.63	−0.77	1.71	.458	0.217
Group×T_2_	2.44	0.66	1.15	3.73	<.001	0.789

Secondary Outcome: Insight Into Illness (SAI)						
EG (*n*=61)			CG (*n*=59)			
T_0_	T_1_	T_2_	T_0_	T_1_	T_2_	
*M* (*SD*)	*M* (*SD*)	*M* (*SD*)	*M* (*SD*)	*M* (*SD*)	*M* (*SD*)	
6.26 (2.85)	9.90 (3.03)	9.61 (3.17)	6.51 (2.97)	7.77 (3.90)	7.98 (3.41)	

Effect	*B*	*SE*	95% CI		*p*	*ES*
			Lower	Upper		
Group	−0.42	0.52	−1.45	0.61	.426	
T_1_	1.24	0.48	0.30	2.18	.010	
T_2_	1.40	0.43	0.56	2.24	.001	
Group×T_1_	1.85	0.63	0.62	3.07	.003	0.816
Group×T_2_	2.23	0.67	0.92	3.54	.001	0.645

Secondary Outcome: Positive Symptom						
EG (*n*=61)			CG (*n*=59)			
T_0_	T_1_	T_2_	T_0_	T_1_	T_2_	
*M* (*SD*)	*M* (*SD*)	*M* (*SD*)	*M* (*SD*)	*M* (*SD*)	*M* (*SD*)	
20.82 (3.26)	13.00 (3.39)	11.61 (3.39)	20.47 (4.40)	15.36 (4.11)	13.05 (3.90)	
Effect	*B*	*SE*	95% CI		*p*	*ES*
			Lower	Upper		
Group	0.62	0.63	−0.62	1.86	.326	
T_1_	−4.92	0.66	−6.21	−3.63	<.001	
T_2_	−7.20	0.65	−8.47	−5.93	<.001	
Group×T_1_	−2.52	0.83	−4.14	−0.90	.002	0.738
Group×T_2_	−1.85	0.85	−3.51	−0.19	.029	0.487

*Note*. ES = effect size; T_0_ = baseline; T_1_ = fourth session after intervention interim analysis; and T_2_ = postintervention.

Covariates = group, time, sex, diagnosis, antipsychotics, type of medicine, age, years of education, duration of illness, marital status, working status, treatment setting, living alone, internalized stigma, chlorpromazine _(100 mg)_ equivalents.

#### Primary Outcome: Medication Adherence Rating Scale (MARS) Score

After controlling for the effects of diagnosis, antipsychotic medication, medication type, age, years of education, marital status, disease duration, employment status, treatment setting, living situation, internalized stigma, and chlorpromazine (100 mg) equivalent dosage as covariates, the average MARS score decreased significantly in the CG at both T_1_ (*B*=−2.01, *p*<.001) and T_2_ (*B*=−1.86, *p*<.001). The GEE analysis revealed no significant between-group improvement at T_1_ (*p*=.281) and a more significant improvement at T_2_ over T_0_ in the EG compared with the CG (*B*=2.56, *p*<.001), with an effect size of 0.912.

A subgroup analysis was performed using GEEs to explore possible differences in the intervention effects between participants from acute wards and outpatient departments. The interaction between group and time revealed the EG participants in both subgroups improved more significantly in terms of MARS score at T_2_ than their CG counterparts.

#### Secondary Outcome: The Schedule for the Assessment of Insight (SAI)

After controlling for the 13 covariates, the average SAI scores for the CG improved significantly at both T_1_ (*B*=1.24, *p*=.010) and T_2_ (*B*=1.40, *p*=.001). The EG showed greater improvement in insight into illness scores, with a 1.85-point greater increase than the CG at T_1_ (*B*=1.85, *p*=.003) and 2.23-point greater increase at T_2_ (*B*=2.23, *p*=.001). The effect sizes were 0.816 and 0.645, respectively.

#### Secondary Outcome: Positive and Negative Syndrome Scale (PANSS) Score

After controlling for the 13 covariates, the positive symptom subscale score improved significantly for the CG at T_1_ (*B*=−4.92, *p*<.001) and T_2_ (*B*=−7.20, *p*<.001). Compared with the CG, the EG showed a 2.52-point greater increase (*p*=.002) greater than CG at T_1_, with an effect size of 0.738. At T_2_, the score of the EG had improved by 1.85 points (*p*=.029) more than the CG, with an effect size of 0.487.

## Discussion

### Effectiveness of Motivational Interview-Based Cognitive Therapy (MIBCT)

The results of this study reveal that medication adherence improved significantly among those participants who had received the MIBCT intervention (especially among those hospitalized) compared with those receiving routine care. For the EG, the average MARS score increased from 5.38 at baseline (T_0_) to 7.00 at the first assessment (T_1_), remaining stable at T_2_, indicating the lasting impact of MIBCT on medication adherence. MIBCT increases intrinsic motivation and behavioral change, with MI helping patients address their ambivalence about change through empathetic guidance (Miller & Rollnick, 2023). The findings of prior research show MI effectively increases medication motivation in patients with schizophrenia, leading to better adherence (Dobber et al., 2018). Moreover, the cognitive therapy incorporated in MIBCT targets and modifies distorted beliefs about medications and illnesses, improving patient understanding regarding these aspects (Iarussi, 2020). The findings of this study highlight the combined effects of MI and cognitive therapy on fostering patient treatment adherence intentions and abilities.

The decline in adherence at T_1_ followed by its increase to a level above baseline at T_2_ may be attributed to several factors. First, some of the participants came from acute wards and had yet to establish medication-adherence-related knowledge and behaviors, leading to lower scores at T_1_. Importantly, medication adherence is a dynamic process that requires ongoing monitoring. Second, research by Kim et al. (2020) based on the Clinical Antipsychotic Trials of Intervention Effectiveness (CATIE) trial revealed the relationship between illness awareness and medication adherence as nonlinear, with nonadherence rates increasing over time. Finally, the MARS used in this study may have resulted in artificially high adherence scores at T_0_ due to reminders from nursing staff, leading to the observed decline at T_1_. These findings highlight the importance of clinical insight in managing schizophrenia effectively.

The subgroup analyses in acute care and outpatient settings revealed medication adherence did not improve after four MIBCT sessions but did improve significantly after eight sessions. This finding supports Schulz et al. (2013), who reported that short-term interventions are often ineffective, and Chien et al. (2024), who reported on the benefits of longer-term interventions.

The findings of this study confirm that, after eight MIBCT sessions, medication adherence improved across clinical settings. Adherence to prescribed medication is crucial for managing schizophrenia, with research showing that more than half of patients fail to follow their medication regimens after hospital discharge (Vega et al., 2021) and in community settings (Karabulut & Uslu, 2024). These results emphasize the importance of integrating MIBCT into treatment and current medication management strategies.

This study, which included eight sessions of MIBCT conducted over a 6–8 month period, yielded a postintervention medication adherence effect size of 0.912. According to Fritz et al. (2012), a sample size of 40 participants per group is sufficient to detect an effect size of 0.61, which is considered a moderate effect. Thus, the MIBCT used in this study may be interpreted as moderately improving medication adherence and clinical insight. This improvement was largely achieved through the two motivational-interview interventions that focused on collaboratively setting treatment goals, establishing family and health care support systems, and addressing obstacles. The sessions included open discussions to clarify medication benefits, strategies for managing side effects, and reward systems to increase treatment acceptance.

Furthermore, the findings revealed that the efficacy of clinical insight was greater in the interim analysis (T_1_) and postintervention analysis (T_2_) than in the motivational-based interviews, which were grounded in interpersonal relationship theory conducted by Köktaş et al. (2023), with respective effect sizes of 0.32 at 6 weeks postintervention and 0.78 at 3 months postintervention. This was attributed to the design focus of the eight sessions of MIBCT integrated with motivational interviewing, which aimed to examine disease and medication experiences and coping strategies guided by a clinical therapeutic perspective. The focus was on increasing participant awareness of their mental illness and intention to engage in treatment to ensure timely help-seeking and improvement in their clinical insight and disease management capabilities.

Finally, a significant difference was noted in the secondary outcome measure of positive symptoms, with effect values of 0.738 at T_1_ and 0.487 at T_2_. This finding indicates that at T_1_ the approach had achieved nearly moderate efficacy. Compared with the study by Chien et al. (2019) in which the EG’s baseline value for positive symptom assessment decreased from a mean of 18.02 to 14.65 at the 6-month follow-up, in this study, the positive symptoms of the EG decreased from 20.82 (*SD*=3.26) at T_0_ to 11.61 (*SD*=3.39) at T_2_. This improvement is attributable to the inclusion in the fifth, sixth, and eighth interventions of measures for recognizing and coping with psychotic symptoms. Therefore, the observed improvement in psychotic symptoms among EG participants in this study was relatively significant.

The attrition rate in this study was 30%, which was higher than the findings of a meta-analysis by Li et al. (2023) in Taiwan (2.5%–4.5%). This is attributable to most of the participants in that analysis being drawn from community centers and outpatient clinics. In contrast, the participants in this study were recruited from acute wards or recently discharged from outpatient clinics, and had more severe symptoms at baseline than the participants in Li and Hsieh’s trials. In addition, in the three studies included in their meta-analysis, patients had a more positive attitude toward antipsychotics, which led to better medication adherence and greater stability. However, according to the findings of a randomized controlled trial conducted by the Canadian scholars Au-Yeung et al. (2023), the attrition rate reached as high as 48% among 96 patients from five psychiatric outpatient clinics participating in psychoeducation and cognitive therapy. The reasons for dropout included hospitalization, unwillingness to participate in the trial, dissatisfaction with the randomly assigned group, and lower years of education, which further explain the high attrition rate observed in this study.

### Strengths and Limitations

In this study, motivational interviewing was integrated with cognitive therapy to improve medication adherence among the target patient group. Ambivalence about medication was addressed, patient understanding of their illness was improved, and negative beliefs surrounding both were changed. Despite the high attrition rate, the results revealed that the eight sessions of MIBCT delivered over a 24–32-week period effectively improved medication adherence and illness awareness and reduced positive symptoms. Notably, research has shown that 58.2% of patients stop taking their medication within 6 months of discharge, often leading to relapse and rehospitalization. Thus, this intervention warrants broader implementation (Vega et al., 2021).

The study was limited to patients within the schizophrenia spectrum. Thus, the results may not extrapolatable to the broader population of individuals with severe mental illness. In addition, the sample was drawn from only two psychiatric centers in northern Taiwan. Thus, the sample may not be representative of the community of patients with schizophrenia in Taiwan. Also, the relatively small sample size from acute inpatient settings may introduce potential bias when making inferences. Finally, although the findings revealed an improvement in medication adherence at T_2_, the degree of improvement remained limited compared with baseline. This finding indicates the 6–8-month intervention may be insufficient to achieve the desired effect, especially when patients do not receive adequate support postintervention, potentially leading to medication discontinuation and relapse. Therefore, in line with the recommendation of Hong Kong scholars Chien et al. (2019), more than 1 year may be necessary to effectively improve medication adherence in patients.

The baseline findings of this study indicate the participants were affected by a moderate level of perceived stigma (Ritsher & Phelan, 2004). However, self-stigma has been shown to affect both medication adherence and insight into illness significantly (Alhadidi et al., 2021). Depressive symptoms, a core clinical feature of schizophrenia, may be further aggravated by perceived stigma (Sarraf et al., 2022). Thus, it is crucial to provide cognitive behavioral therapy to patients with poor medication adherence to mitigate self-stigma, particularly among patients whose stigma is closely linked to maladaptive rumination (Chiang et al., 2025).

### Future Directions

This study did not follow-up after 32 weeks. Patients in the psychiatric acute ward experienced greater effect sizes than their outpatient clinic peers. Future research should expand the acute ward participant group because these patients often face rehospitalization due to stopping medication. The follow-up period should also be extended to at least 1 year, with consideration for even longer (2, 5, or 10 y) longitudinal follow-up. Future studies may also consider extending the scope of MIBCT to include mood-related outcomes.

### Conclusions

The program implemented in this study provides patients who had previously taken medication irregularly with eight sessions of motivational interview-based cognitive therapy. The results indicate MIBCT can effectively improve medication adherence, insight into illness, and positive symptoms in patients with schizophrenia spectrum disorders.

### Implications for Nursing Practice

MIBCT should be provided to patients with repeated hospitalization within a 6-month period to improve their medication adherence, illness awareness, and psychotic symptoms management capabilities. Community mental health nurses should gain proficiency in MIBCT and, when identifying patients with medication nonadherence in the community, implement MIBCT to promote medication adherence and illness awareness to reduce the recurrence of patient symptoms. This study offers a reference for mental health nurses for supporting patients in terms of medication adherence efficacy, illness understanding, and symptom maintenance.

## Supplementary Material
