# Supplementary material for: Effects of Motivational Interview-Based Cognitive Therapy in Patients With Schizophrenia: A Multicenter Randomized Controlled Trial
Source: J Nurs Res. 2026 Aug 13;34(4):e465. doi: 10.1097/jnr.0000000000000761 (PMC13465704; doi:10.1097/jnr.0000000000000761)
Supplement: Supplementary file 1 [file jnr-34-e465-s001.docx]

**Appendix**

Motivational Interviewing-Based Cognitive Therapy

| Goals | Domain/Influential Factors | Strategy | Interventions | Outcome |
| --- | --- | --- | --- | --- |
| Provide comprehensive insights into illness of schizophrenia and the corresponding pharmacological interventions, concurrently facilitating the instruction of self-management skills. | 1.1 Individual domain: therapeutic effects and experience of taking antipsychotics 1.2 Psychological domain: patient attitude toward illness and medication   1.3 Social and environmental domain: therapeutic alliances | Session 1.  Help the patient to review their medication experience  (Motivational Interviewing [MI]: Engaging) | 1-1-1 Understand the patient’s medication experience through open-ended questions. Example: *"Can you tell me what it has been like for you taking your medication every day*?*"* 1-2-1 Employing patient-centered principles, execute an interview. Example: *"I’d like to understand how you feel about your treatment, and your thoughts truly matter in determining what works best for you*.*"* 1-3-1 Use therapeutic communication skills and empathy to build therapeutic alliances. Employing reflective listening, such as *"It sounds like the medication changes how you feel in a way that’s uncomfortable*.*"* | 1. Schedule for the Assessment of Insight (SAI) |
|  | 2.1 Individual domain: therapeutic effects and side effects of the antipsychotics   2.2 Psychological domain: insight into illness, patient attitude toward illness, and medication | Session 2.  Assist the patient in engaging in self-management practices in the context of antipsychotic medication.  (MI: Focusing) | 2-1-1 Assess the patient’s knowledge and attitudes toward taking antipsychotics (including barriers and facilitators). Example: *" It sounds like you understand the purpose of the medication, but you’re also experiencing side effects that make it harder to take. What kinds of things help you remember to take it, even when it’s difficult?*" 2-2-1 Use the scale score to ascertain the patient's disposition toward treatment, employing a 0 to 10-point scoring system to systematically appraise the patient's attitude toward the therapeutic effects and potential side effects of the antipsychotics. Example: *" On a scale from 0 to 10, where 0 indicates no effect at all and 10 indicates maximal effectiveness, how would you rate the extent to which your current medication has improved your symptoms? What factors influenced your choice of this score (e.g., an 8)?"* | 1. Schedule for the Assessment of Insight (SAI) |
|  | 3.1 Individual domain:  medication side-effects,  medication efficacy,  medication duration  3.2 Psychological domain: insight into illness | Session 3.  Deliver information specifically related to the disease and medication in the context of schizophrenia.  (MI: Evoking) | 3-1-1 To understand and address psychological ambivalence, enhance awareness of cognitive biases, and restructure negative beliefs about medication and illness, the intervention included psychoeducation on the effects and side effects of antipsychotic medications. Additionally, medication pictograms and individualized medication pouches were integrated to facilitate the patient's understanding of the treatment.  3-1-2 Engage in a comprehensive dialogue with the patient to explore the underlying factors contributing to medication discontinuation. Examples: *"What factors have contributed to your decision to stop or adjust the dosage of your medication? On one hand, you’re uncertain about the effectiveness of the medication; on the other hand, you’re concerned that psychiatric medications might be having some physical side effects or making you feel uncomfortable—is that right?"*  3-2-1 Provide disease-related knowledge on schizophrenia. For instance, *illustrated educational leaflets may be used to enhance patients’ understanding of the illness, including its etiology, symptoms, treatment, and management.* | 1. Schedule for the Assessment of Insight (SAI)  2. Medication Adherence Rating Scale (MARS)  3. Positive and Negative Syndrome Scale  (PANSS) |
| Clarify the patient's goals for change and assist in formulating a plan for taking antipsychotics. | 4.1 Individual domain: medication side-effects, medication efficacy, and medication duration  4.2 Social and environmental domain: therapeutic alliances | Session 4.  Discuss the accomplishment of medication goals and actions in the past 2 weeks.  (MI: Planning) | 4-1-1 Assist patients in collaboratively developing medication goals and strategies. Using a patient-centered approach, support patients in formulating personalized medication objectives, such as reducing the frequency of hallucinations, improving daily functioning, and minimizing the burden of side effects.  4-1-2 Help the patient record the reasons for maintaining or discontinuing medication. *For instance, encouraged to systematically document their motivations and considerations regarding the continuation or discontinuation of antipsychotic medication. These reflections should encompass both positive aspects, such as symptom improvement and the prevention of hospitalization, as well as negative experiences, including the manifestation of adverse side effects. Engaging in this process may promote a more comprehensive understanding of treatment dynamics and facilitate increased patient engagement, which could, in turn, improve adherence to prescribed therapeutic regimens.*  4-2-1 Use homework to help the patient review how he/she has reached the medication goals. For instance, using a structured worksheet to guide patients in observing and recording daily changes following medication—such as symptoms, sleep patterns, mood, or side effects—can enhance their self-monitoring skills. This approach increases awareness of treatment effects and medication outcomes, which can then be reviewed and adjusted in subsequent sessions. In turn, this process may strengthen treatment motivation and promote continued engagement in care. | 1. Schedule for the Assessment of Insight (SAI) 2. Medication Adherence Rating Scale (MARS) |
|  | 5.1 Individual domain:  medication side-effects, medication efficacy,  5.2 Psychological domain: self-efficacy for medication management, insight into illness, patient attitude toward illness and medication and symptom severity  5.3 Social and environmental domain: family support | Session 5.  Reduce barriers to medication adherence and develop medication management plans  (MI: Evoking, Planning) | 5-1-1 Identify barriers and facilitators for taking antipsychotics. Example: *"What are some factors that might help you continue taking your medication? And what are some factors that might make it more difficult?"*  5-1-2 Facilitate the patient in conducting a thorough assessment of the pros and cons, with a specific emphasis on the benefits of the treatment. Example: *"Let’s list together the pros and cons of taking medication. At this point, which side do you feel is outweighing the other*?"  5-2-1 Help the patient identify symptoms, provide reality ideas, develop discrepancies, and enable patients to understand the need for treatment. Example: *"You mentioned facing some challenges at work, and at the same time, you’ve expressed a desire to return to your job. Do you feel that your current situation might affect your ability to reach that goal?"*  5-2-2 Discuss the association between the reduction and discontinuation of medication and the potential relapse of symptoms. Example: *"For many people, symptoms tend to return within a few weeks or months after stopping medication. Do you think your previous hospitalization might have been related to something like that?"*  5-3-1 Facilitate the collaborative engagement of both the patient and their family member in jointly formulating and implementing medication management plans. Example: *"Would you be open to having your mother involved in our medication management plan? She could help remind you to take your medication or keep a record, and help us observe any side effects."* | 1. Schedule for the Assessment of Insight (SAI) 2. Medication Adherence Rating Scale (MARS) 3. Positive and Negative Syndrome Scale   (PANSS) |
| Evaluation of patient medication adherence and insight into illness. | 6.1 Psychological domain: self-efficacy for medication management, insight into illness, patients’ perceived stigma, severity of psychotic symptoms | Session 6. Understand patient treatment beliefs  (MI: Evoking) | 6-1-1 Present successful and unsuccessful cases to enable the patient to acquire valuable insights and successfully modify behaviors through experiential learning from these cases. Example: "*We had a patient who was able to return to work after staying on his medication. However, after stopping it on his own for two weeks, he started experiencing auditory hallucinations and insomnia again. Later, he said he wished someone had reminded him about the risks. What are your thoughts on this story?*"  6-2-1 Promote patient engagement in recognizing and identifying the characteristic relapse of disease. Example: " Before your last hospitalization, did you observe any changes in your sleep quality, thoughts, or mood?"  6-2-2 Discuss the chronic disease nature of mental diseases and compare them with other chronic diseases (diabetes and hypertension). Example: *"Psychiatric disorders, much like chronic conditions such as diabetes or hypertension, often require consistent medication to maintain stability. The medications we use help stabilize your thoughts and emotions. It’s not about a lack of effort — it’s that the brain, just like other organs, sometimes needs long-term care."* | 1. Schedule for the Assessment of Insight (SAI) 2. Medication Adherence Rating Scale (MARS) 3. Positive and Negative Syndrome Scale   (PANSS) |
|  | 7.1 Individual domain: medication side-effects, medication efficacy, and medication duration  7.2 Psychological domain: patients’ perceived stigma and self-efficacy  7.3 Social and environmental domain: social support | Session 7 Reduce social stigma and strengthen social support systems  (MI: Evoking, Planning) | 7-1-1 Use standard terms to explain diseases and drugs to reduce stigma. Example: *"This type of medication is called an antipsychotic. Its function is to help stabilize the way the brain transmits signals — similar to how diabetes medications help regulate blood sugar levels."*       7-1-2 Affirm the decisions made by the patient and reinforce their sense of self-efficacy in taking antipsychotics. Example: *"You did a great job! This time, you proactively recorded the side effects and shared them with us. I see this as a very responsible action, showing that you genuinely care about your own health*."       7-2-1 Continuously monitor medication management plans. Example: *"Previously, we collaboratively established a strategy for medication adherence reminders. How effective has it been for you over the past week? Do you feel any modifications are necessary?"*       7-3-1 Evaluate the familial and social support resources available to the patient. Example: *"Who do you usually talk to about your condition?"; "When you’re feeling distressed, who do you tend to confide in?"; "Is there anyone who reminds you to take your medication?"* | 1 Schedule for the Assessment of Insight (SAI) 2 Medication Adherence Rating Scale (MARS) |
|  | 8.1 Psychological domain: insight into illness, patient attitude toward illness and patient attitude toward medication  8.2 Social and environmental domain: social support | Session 8 Strengthen social support systems and summary medication management program  (MI: Planning) | 8-1-1 Engaging patients in a collaborative review of their medication experience, including the perceived efficacy, challenges encountered during treatment (such as side effects or forgetfulness), and positive outcomes like symptom improvement, can help foster a more constructive understanding of their treatment journey. This reflective process not only informs future medication planning but also strengthens the patient’s insight into illness, enhances self-management capabilities, and promotes the development of self-monitoring skills. For example, *"Looking back on your recent experience with medication, were there aspects you found particularly effective? Or were there any side effects that you feel need to be addressed?"* 8-1-2 Evaluation of insight into illness and medication adherence. For example, structured assessment tools—such as SAI or MARS—may be employed to systematically evaluate the patient’s level of insight into their illness and the degree of adherence to prescribed treatment. These tools provide concrete information on the patient’s awareness of their condition and their acceptance of pharmacological interventions. 8-2-1 Encourage patients to participate in community-based psychiatric rehabilitation or programs. such as day care, sheltered workshops, or community rehabilitation centers to help them establish a stable daily routine. Participation in such programs not only offers emotional support but also promotes peer connections and the development of social skills, thereby strengthening the patient’s overall support system. | 1. Schedule for the Assessment of Insight (SAI) 2. Medication Adherence Rating Scale (MARS) 3. Positive and Negative Syndrome Scale   (PANSS) |

*Note.* The manual used in this study was authorized by Chien et al. (2016) and Gray et al. (2010).
